# Reduced Expression of Sprouty1 Contributes to the Aberrant Proliferation and Impaired Apoptosis of Acute Myeloid Leukemia Cells

**DOI:** 10.3390/jcm8070972

**Published:** 2019-07-04

**Authors:** Valentina Rosso, Cristina Panuzzo, Jessica Petiti, Sonia Carturan, Matteo Dragani, Giacomo Andreani, Carmen Fava, Giuseppe Saglio, Enrico Bracco, Daniela Cilloni

**Affiliations:** 1 Department of Clinical and Biological Sciences, University of Turin, 10043 Turin, Italy; 2 Department of Oncology, University of Turin, 10043 Turin, Italy

**Keywords:** Sprouty1, acute myeloid leukemia (AML), FoxO3a

## Abstract

In most of the acute myeloid leukemia patients there is an aberrant tyrosine kinase activity. The prototype of Sprouty proteins was originally identified in *Drosophila melanogaster* as antagonists of Breathless, the mammalian ortholog of fibroblast growth factor receptor. Usually, SPRY family members are inhibitors of RAS signaling induced by tyrosine kinases receptors and they are implicated in negative feedback processes regulating several intracellular pathways. The present study aims to investigate the role of a member of the Sprouty family, Sprouty1, as a regulator of cell proliferation and growth in patients affected by acute myeloid leukemia. Sprouty1 mRNA and protein were both significantly down-regulated in acute myeloid leukemia cells compared to the normal counterpart, but they were restored when remission is achieved after chemotherapy. Ectopic expression of Sprouty1 revealed that it plays a key role in the proliferation and apoptotic defect that represent a landmark of the leukemic cells. Our study identified Sprouty1 as negative regulator involved in the aberrant signals of adult acute myeloid leukemia. Furthermore, we found a correlation between Sprouty1 and FoxO3a delocalization in acute myeloid leukemia (AML) patients at diagnosis, suggesting a multistep regulation of RAS signaling in human cancers.

## 1. Introduction

Acute myeloid leukemia (AML) develops from the malignant transformation of immature hematopoietic cells through a complex multistep process that requires the cooperation of different genetic alterations [1]. In most of the AML patients, there is an aberrant tyrosine kinase (TK) activity, which results in an impaired differentiation, altered cell growth, and apoptosis defect. Sprouty proteins inhibit the RAS pathway, frequently constitutively active in many human tumors, contributing robustly to cells aggressiveness and invasiveness [2,3,4,5].

The Sprouty family proteins were initially identified in *Drosophila melanogaster* as antagonists of receptor tyrosine kinase (RTK) signaling during different morphogenetic processes, including the development of the trachea, the eye, the wing, and other tissues [6,7,8,9,10,11].

The biological functions of the Sprouty proteins have been attributed to its conserved motifs: all Sprouty proteins share a characteristic Cys-rich C-terminus domain (SPRY domain), which is believed to be indispensable for their function [12,13].

Sprouty proteins have been implicated in the regulation of the biological processes responsible for tumor growth, development, and metastasis, including cell proliferation, migration, invasion, and survival. The downregulation of Sprouty family members has been detected in a number of solid cancers such as breast, prostate, and renal cell carcinoma [3], as well as in leukemia [14].

Experimental evidence showed that depending on the cellular context, Sprouty proteins can paradoxically act either as negative or positive regulators of tumor progression [15,16,17,18]. The presence of mutations on the RAS cascade has also been shown to be an important determinant of the Sprouty’s deregulated action [19]. It has also been reported that a cross-talk between Forkhead box O3 (FoxO3a) and Sprouty family proteins occurs [20,21,22].

FoxO3a belongs to the family of forkhead transcription factors, which are characterized by the presence of a DNA binding domain highly conserved called “forkhead box” [23]. Human forkhead proteins are represented by 4 members: FoxO1, FoxO3a, FoxO4, and FoxO6, and are normally present in an active form in the nucleus. The FoxO proteins have partially overlapping functions: their target genes are involved in processes, such as cell cycle arrest [24,25,26], DNA repair [25,27], cell differentiation [28], apoptosis [29,30,31], and homeostasis of the hematopoietic system through the regulation of the hematopoietic stem cell (HSC) compartment [32]. FoxO family operates under the negative control of Akt: in response to the binding of growth factors (e.g., insulin) to their membrane receptors, the phosphatidylinositol 3-kinase (PI3K) is activated. The activated Akt in turn then phosphorylates FoxO proteins, resulting in the inactivation of these transcription factors and in their translocation from the nucleus to the cytoplasm. Moreover, in breast cancer, the cytoplasmic localization of FoxO3a is correlated with poor survival [33]. Similarly, in leukemia patients FoxO3a phospho-status correlates with some clinical features, such as the percentage of bone marrow (BM) blasts, absolute peripheral blood-(PB) and white blood cells-(WBCs) count, primary resistance, early relapse, and overall survival [34], thus suggesting a pivotal role of FoxO proteins in cancer cells.

In this study, we investigated the role of Sprouty1 as the regulator of cell proliferation and growth in adult patients affected by AML and we studied the correlation between low Sprouty1 expression and FoxO3a delocalization in AML at diagnosis, suggesting a multistep regulation of RAS signaling in human cancers.

## 2. Materials and Methods

### 2.1. Patients and Cell Lines

Informed consents were obtained in accordance with the Declaration of Helsinki. Samples were collected and analyzed under San Luigi Hospital internal institutional ethical committee–approved protocol (approval number 201/2014). Eighty-two BM and 8 PB specimens from AML patients at diagnosis, 15 PB from AML patients after therapy and 16 BM and 18 PB from healthy subjects were collected. All the patients have been previously characterized at the cytogenetic level by conventional karyotyping and screened by reverse transcriptase-PCR for the presence of the most frequent fusion transcripts. Mutations or internal tandem duplication of both Fms Related Tyrosine Kinase 3 (FLT3) and of Nucleophosmin 1 (NPM1) genes were also characterized. Acute promyelocytic leukemia samples were excluded from the study.

The human Kasumi-1 cell line was purchased from ATCC and cultured in RPMI-1640 supplemented with 20% fetal bovine serum (FBS), 500 U/mL penicillin, and 0.5 mg/mL streptomycin. Cells were cultured at 37 °C in a humidified atmosphere flushed with 5% CO_2_.

### 2.2. RNA Extraction and Quantitative Real-Time PCR (qRT-PCR)

Total RNA was extracted using TRIzol Reagent (Ambion, Thermo Fisher Scientific, Waltham, Massachusetts, MA, USA), according to the manufacturer’s instructions. 1 µg of total RNA was reverse transcribed using random hexamers as primers in a final volume of 25 µL. For Sprouty1 mRNA quantification, specific assays (assay ID for ABL Hs00245445_m1, and Hs00544790_m1 for Sprouty1—Applied Biosystems, Thermo Fisher Scientific, Massachusetts, MA, USA) were used according to the manufacturer’s instructions. The analysis was performed in triplicate. The Sprouty1 Cts obtained by qRT-PCR were normalized with respect to the Ct of ABL and expressed as 2^−ΔΔCt^. Universal human references RNA (Stratagene, San Diego, CA, USA) was used to calibrate the assay.

### 2.3. Cells Lysis

For total cell extracts, cells were washed with ice-cold phosphate-buffered saline (PBS) and lysed with RIPA buffer on ice (10% glycerol; 1% Triton X-100; 20 mM Hepes pH 7.4; 5 mM EDTA pH 7.2; 150 mM NaCl) supplied with protease and phosphatase inhibitors (1 mM Na_3_VO_4_, 1 mM PMSF, 2 µg/mL leupeptin, 2 µg/mL aprotinin, 2 µg/mL pepstatin). For nuclear and cytoplasmatic extracts, cells were washed with ice-cold PBS and incubated on ice in 600 µL of cytosolic lysis buffer (10 mM Hepes pH 7.9; 10 mM KCl; 0.1 mM EDTA; 0.5% NP40; 1 µg/mL leupeptin, 1 µg/mL aprotinin, 1 µg/mL pepstatin; 1 mM Na3VO4, 100 µg/mL PMSF). After 30 min, nuclei were separated by centrifugation at 3000× *g* for 10 min and the supernatants collected (cytoplasmic fraction). Nuclei pellets were resuspended in 100 µL of nuclear lysis buffer (20 mM Hepes pH 7.9; 400 mM KCl; 1 mM EDTA; 1 mM EGTA; 1 mM DTT; 10% glycerol; 1 µg/mL leupeptin, 1 µg/mL aprotinin, 1 µg/mL pepstatin; 100 µg/mL PMSF) and incubated on ice for 20 min with vigorous mixing. The nuclear lysates were further clarified by high-speed centrifugation.

### 2.4. Western Blot Analysis

Seventy µg of total proteins were loaded and run onto 10% Sodium Dodecyl Sulphate - PolyAcrylamide Gel Electrophoresis (SDS-PAGE) and transferred to PVDF (Bio-Rad, Hercules, California, CA, USA) membranes. Membranes were blocked in TBS (Tris-HCl pH 7.4, 150 mM NaCl) plus 5% BSA for 1 h at room temperature (RT) and then decorated with appropriate antibodies (Sprouty1 sc-365520, Tubulin sc-23948, and phospho-Akt1/2/3 (Thr308) sc-16646, Santa Cruz Biotechnology; TATA Binding protein (TBP) MA1-189 and Vinculin MA5-11690, Sigma-Aldrich; Foxo3a #2497, p44/42 MAPK (ERK1/2) #4696s, phospho-p44/42 MAPK (T202/Y204) #4377s and Akt (pan) (C67E7) #4691s, Cell Signaling) in PBS-Tween 0.2% overnight at 4 °C. Membranes were then washed with PBS-Tween 0.2% three times for 15 min each, incubated with appropriate peroxidase-linked secondary antibody (Santa Cruz Biotechnology) for 1 h at RT and washed again in PBS-Tween 0.2%. Specific binding was detected using an enhanced chemiluminescence system (Clarity Western ECL Substrate #170-5061, Bio-Rad).

### 2.5. Immunofluorescence Assay

Cytospins were prepared using BM cells from AML patients at diagnosis or in remission phase and Kasumi-1 cell line. Cells were fixed with 4% PFA, permeabilized, and blocked for 45 min. Then, cells were incubated for 2 h at RT with polyclonal anti-Sprouty1 or polyclonal anti-FoxO3a antibodies. The detection of proteins was obtained by incubation for 30 min with secondary antibodies. Cells were then incubated for 5 min with propidium iodide for nuclear staining and analyzed with confocal scanning microscope (LSM 5110; Carl Zeiss MicroImaging Inc., Oberkochen, Germania). Images were captured using a 63× objective. The fluorescent signal was measured by image processing (LSM800) and analyzed in Java (Image J) program https://imagej.nih.gov/ij/.

### 2.6. Plasmid Construction and Transfection

pCGN-Sprouty1 and pECE-FoxO3a (kindly donated by Prof. P.P. Pandolfi) vectors were used for transient transfection of Kasumi-1 cells by FuGENE-6 (Roche Applied Science, Basilea, Svizzera), according to the manufacturer’s instructions.

The simultaneous transfection with pEGFP-C2 vector alone allowed one to check the transfection efficiency after 48 h.

### 2.7. Proliferation and Apoptosis Assays

Cell growth was evaluated by MTT assay (Cell Proliferation Kit I (MTT), Sigma-Aldrich, St. Louis, Missouri, MI, USA), according to the manufacturer’s instructions. Experiments were performed in triplicate. Apoptosis was evaluated by flow cytometry measuring annexin staining. Briefly, cells were washed once with PBS 1× and incubated for 15 min with fluorescein isothiocyanate (FITC)-conjugated annexin V and propidium iodide (Annexin V-FITC Apoptosis Detection Kit, Immunostep, Salamanca, Spain). After incubation, cells were analyzed by flow cytometry. For all samples, at least 100,000 events were acquired. BD CellQuest software (BD Biosciences, New Jersey, NJ, USA) was used for data analysis.

### 2.8. Colony Growth Assay

Kasumi-1 cells, transfected with pCGN-Sprouty1 and pCGN empty vector, were plated in RPMI-Soft Agar to test their clonogenic ability. Appropriate control samples were plated for each experiment. After 2 weeks, cells were stained with Crystal Violet, visualized, and counted by Infinity Analyze 3 camera and processed by Lumenera software.

### 2.9. Statistical Analysis

Statistical analyses were performed using the two-tailed unpaired student’s *t*-test. All the analysis with confidence level major of 95% are indicated like significant and marked as followed: * *p* ≤ 0.05; ** *p* ≤ 0.01; *** *p* ≤ 0.001.

## 3. Results

### 3.1. Sprouty1 mRNA and Protein Are Both down Regulated in AML Patients at Diagnosis

**Figure 1:**
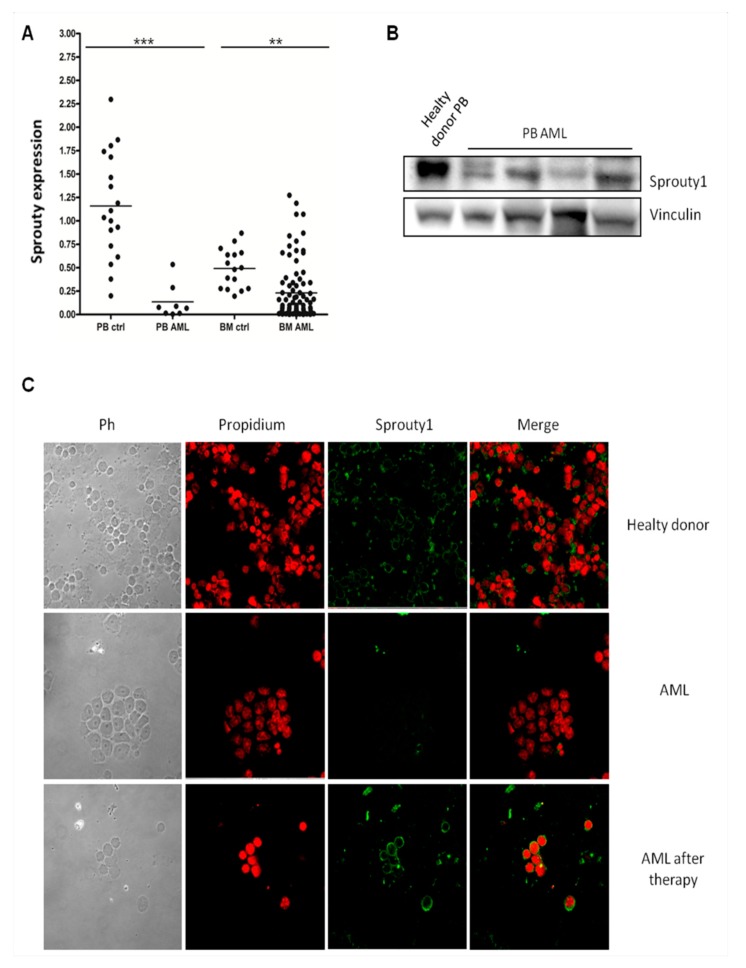
(**A**) *Sprouty1* gene expression was assayed by qRT-PCR in BM and PB derived from both AML patients and normal subjects. The quantity is expressed as 2^−ΔΔCt^ after normalization with *Abl* housekeeping gene (** *p* ≤ 0.01 and *** *p* ≤ 0.001). (**B**) Western blot performed with anti Sprouty1 antibody on total protein derived from PB of four representative AML cells and one PB of a healthy donor. Vinculin is used as normalizer. (**C**) Immunofluorescence staining assay performed on cytospun BM cells of AML or control samples. The green signal corresponds to Sprouty1 while red propidium iodide is used to stain nuclei. BM, bone marrow; PB, peripheral blood; AML, acute myelocytic leukemia.

We initially analyzed the Sprouty1 gene expression by quantitative Real-Time PCR (qRT-PCR) in BM and PB samples collected from 90 AML patients at diagnosis and 34 healthy subjects. The sprouty1 transcript is significantly decreased in both BM and PB of AML patients when compared to healthy subjects (Figure 1A). The median value of transcript expressed as 2^−ΔΔCt^ is 0.3 in BM from AML patients compared to 0.5 in BM from healthy subjects (*p* ≤ 0.01) and 0.18 in PB from AML patients compared to 1.15 in PB from healthy subjects (*p* ≤ 0.001). There is no significant difference in Sprouty1 gene expression according to the French-American-British (FAB) subtypes or according to different chromosomal translocations or FLT3 mutations. Subsequently, we investigated Sprouty1 protein amount and localization in primary leukemic cells derived from AML patients by Western blot and immunofluorescence assay. Western blot of four representative patients and one control showed the presence of the 35 kDa immunoreactive protein Sprouty1 in the sample derived from a healthy donor. By contrast, the protein was barely detectable in leukemic cells (Figure 1B).

In line with these results, immunofluorescence assay showed that cytoplasm of normal controls was stained brightly by the anti-Sprouty1 antibody, while in AML patients the protein is completely absent (Figure 1C). To further confirm that Sprouty1 downregulation is a specific feature of AML, we analyzed the same patients at the time of complete remission after chemotherapy. Immunofluorescence showed that the intensity and localization of Sprouty1 are completely restored as in control cells (Figure 1C).

### 3.2. Overexpression of Sprouty1 Induces Apoptosis, Inhibits Proliferation and Colonies Growth in Kasumi-1 Cell Line

**Figure 2:**
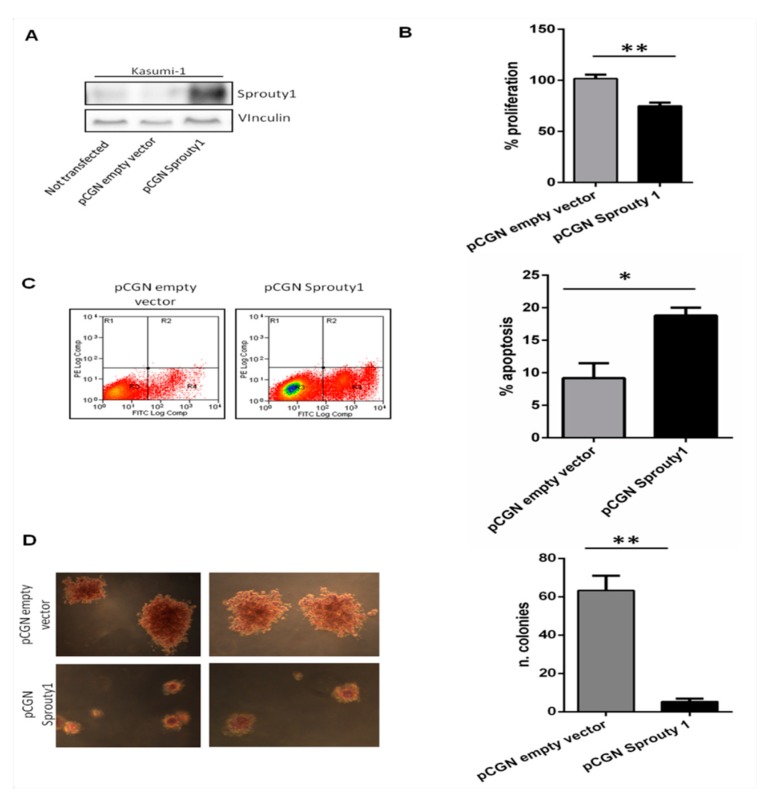
(**A**) Western blot analysis and quantification performed on Kasumi-1 cell lines transfected, respectively, with pCGN empty vector and pCGN-Sprouty1 vector. (**B**) Proliferation assay performed in Kasumi-1 cells transfected with empty or Sprouty1 vector. (**C**) Apoptosis evaluated by flow cytometry after FITC Annexin-V assay on Kasumi-1 cells transfected with pCGN-Sprouty1. (**D**) RPMI-Soft Agar colony assay on Kasumi-1 transfected cells. Representative colonies pictures were captured by Infinity Analyze 3 camera and processed by Lumenera software. All experiments were performed in triplicate.

To investigate the negative role of Sprouty1 in sustaining the leukemic proliferation and favoring apoptosis defect, we transiently overexpressed the Sprouty1 in the Kasumi-1 cell line. After confirming the increased level of Sprouty1 protein in transfected cells (Figure 2A), we conducted proliferation and apoptosis assays. We examined the proliferation activity of transfected Kasumi-1 cells by MTT assay and we observed a significant inhibition of proliferation in cells transfected with pCGN-Sprouty1, with a 30% reduction compared to Kasumi-1 cells transfected with control vector (*p* ≤ 0.01) (Figure 2B).

Sprouty1 overexpression significantly increased the number of apoptotic cells when compared to cells transfected with the empty vector (mean values 18% compared to 10%, respectively, *p* ≤ 0.05) (Figure 2C).

Finally, we evaluated the effect of Sprouty1 on clonal growth in Kasumi-1 cells. Following transfection, cells were seeded in RPMI-Soft Agar for colony assays. Colony growth was strongly inhibited, and size dramatically reduced compared to control cells transfected with empty vector (*p* ≤ 0.01), further demonstrating the role of Sprouty1 in leukemia cell growth (Figure 2D).

### 3.3. FoxO3a Protein Is Delocalized in AML Patients at Diagnosis

**Figure 3:**
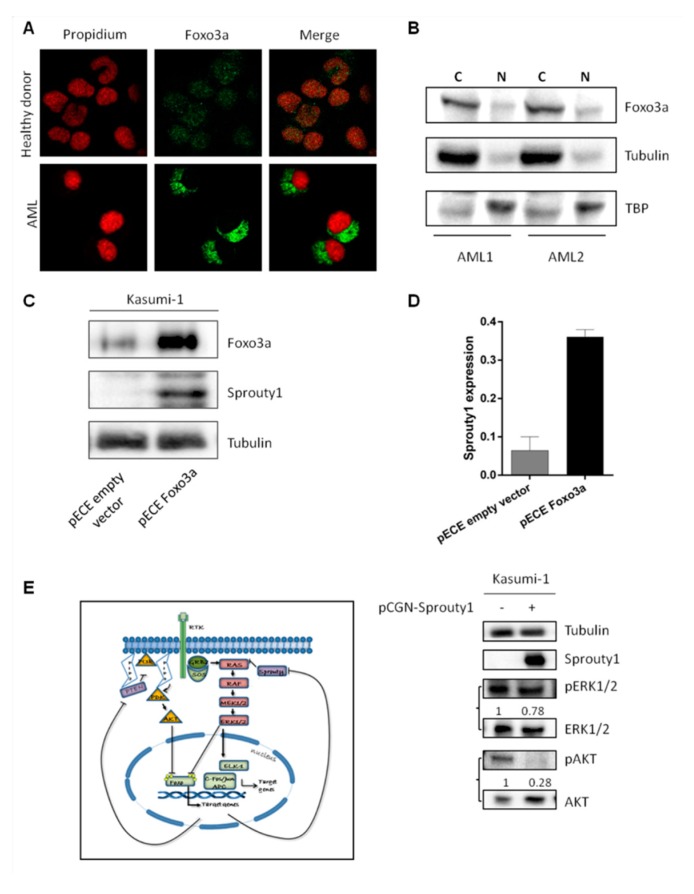
(**A**) Immunofluorescence staining performed with anti-FoxO3a (green signal) on cells derived from control subjects and AML patients at diagnosis. (**B**) Western blot performed with an antibody against FoxO3a on lysates derived from the cytosolic and nuclear fraction of AML patients. (**C**) Western blot of FoxO3a and Sprouty1 in Kasumi-1 cells transfected with FoxO3a or empty plasmids. (**D**) *Sprouty1* gene expression analysis on Kasumi-1 cells transfected with FoxO3a or empty plasmids. The quantity is expressed as 2^-ΔΔCt^ after normalization with *Abl* housekeeping gene. (**E**) Schematic representation of RAS/PI3K pathways and their negative regulation on FoxO3a. Representative western blot showing Sprouty1-dependent regulation of the RAS pathway in Kasumi-1 cells transfected with Sprouty1 plasmid. The relative intensity of each band is shown under the blot as fold change compared to non-transfected control, to which a value of 1 unit was assigned.

In order to investigate the mechanisms leading to impaired Sprouty1 expression in AML patients, we analyzed the transcription factor FoxO3a that is known to be one of its regulator [35]. Immunofluorescence assay performed on primary adult AML cells showed that FoxO3a is exclusively localized within the cytoplasm and it is absent in the nucleus thus suggesting its complete loss of the transcription activity. By contrast, FoxO3a is localized in both cytoplasmic and nuclear compartments from cells harvested from a healthy donor (Figure 3A). This result was confirmed in AML cells by Western blot performed on cytosolic and nuclear lysates, respectively. As shown in Figure 3B, a thick band is observed only in the columns corresponding to cytoplasmic lysates.

To further assess the role of FoxO3a in downregulation of Sprouty1, we ectopically expressed FoxO3a in Kasumi-1 cells. After confirming the increased protein in transfected cells (Figure 3C), we evaluated if FoxO3a could positively regulate Sprouty1 by analyzing its mRNA and protein levels. As shown in Figure 3C,D, both Sprouty1 proteins and mRNA were significantly increased in FoxO3a transfected cells, suggesting a direct cross-talk between these proteins.

AML cells are characterized by sustained Ras activation and its main downstream signaling pathways: MEK/ERK1-2 and PI3K/Akt. Thus, based on this premise, we assessed the phosphorylation status of both ERK1/2 and Akt in Kasumi-1 cells.

Accordingly, our results showed that the overexpression of Sprouty1 negatively regulates the PDK1-dependent AKT phosphorylation (S308) whereas ERK1/2 phosphorylation is only partially inhibited, suggesting a role for Sprouty1 in regulating both signaling pathways with different strengths in Kasumi 1 cells (Figure 3E).

## 4. Discussion

More than a decade ago the genomic and epigenomic landscape of a de novo adult patient affected by AML with a normal karyotype has been characterized [36]. Since then, next-generation sequencing technologies have been largely applied to molecularly characterize adult AML patients with the aim to further improve the prognostic risk assessment relevance [37]. In addition, the study revealed that, from the genomic point of view, AML is an extremely heterogeneous clonal hematopoietic stem cell malignancy, characterized by chromosomal abnormalities, recurrently mutated genes, epigenetic modifications affecting chromatin structure, microRNA deregulations [38]. Surprisingly from these studies, it has emerged that, genomically speaking, AML is a disorder even more multifaceted than previously expected. Beside the genomic heterogeneity, which allowed us to stratify patients on a risk assessment basis, the identification of the mutational signature laid the basis for novel molecular targeted therapies. Indeed, despite nowadays the first line standard protocol for adult AML treatment still forecasts an induction-consolidation regimen with DNA damaging agents (e.g., DNA topoisomerase inhibitors), lately patients are also successfully treated with FLT3 [39] and Isocitrate Dehydrogenase 1/2 (IDH1/IDH2) inhibitors [40]. However, the future development of alternative therapies requires the elucidation of the molecular mechanisms sustaining the leukemic clones.

Depending from the cellular context Ras signaling can lead either to cell growth or development by means of spatial and temporal control and most cancers evade these regulations, including AML-cells, where it represents an unfavorable prognostic marker. Sprouty members are among the most evolutionary conserved Ras signal transduction regulators in animal organisms. In this study, we aimed to assess the role of Sprouty1 in adult AML specimens and cell lines. RAS pathway mutations closely associated with M4/M5 phenotypes [41,42]. Interestingly, germline loss of function mutation of a Sprouty family member, SPRED1, was reported to predispose to AML M5 [43]. We demonstrated that the downregulation of Sprouty1 plays a central role in sustaining the leukemic clone. Sprouty1 mRNA and protein levels were significantly decreased in AML samples when compared to the healthy subjects. Strikingly, the transcription level is restored when remission is achieved after chemotherapy. The role of Sprouty1 in leukemia cell survival and growth is strengthened by our data showing that the Kasumi-1 cells overexpressing Sprouty1 displayed increased apoptosis and inhibited proliferation levels as well as the colonies growth rate. FoxO3a is a potential candidate Sprouty1 transcriptional regulator [44,45]; we analyzed FoxO3a subcellular localization in AML specimens to assess its role in regulating Sprouty1 gene expression. Indeed what we observed was a sharp PI3K dependent FoxO3a cytoplasmic relocalization where it is known to be transcriptionally inactive. Furthermore, activation of ERK has been shown to phosphorylate FoxO proteins, resulting in subsequent MDM2-dependent ubiquitination and protein degradation [46]. Accordingly, our data indicate that the inactivation of FoxO3a might occur either via the RAS/ERK pathway or via the AKT pathway, and which could be responsible for the decline of Sprouty1 with this effect being mainly driven by the PI3K axis. Otherwise, post-transcriptional events FoxO3a related could sustain the low Sprouty1 expression in AML, and in turn, this negative feedback could imply an increase in RAS activity. In AML patients, RAS and PI3K pathways are frequently deregulated or constitutively activated [4,5]. Rather than robust and sustained cell growth, one of the most striking features of AML blasts is their severely impaired differentiation process leading to a prolonged cell survival mainly sustained by the PI3K-AKT signaling axis. Although the Sprouty proteins activities have been largely linked to the MEK-ERK1/2 pathway, it is also known that they might affect the PI3K-AKT pathway. Overall, our data indicate that the Kasumi cell line displayed impaired ERK1/2 and AKT phosphorylation status as a consequence of Sprouty1 overexpression. Nonetheless, we noticed that the phosphorylation decline was more pronounced for AKT. To some extent, these data are in line and might explain the moderate effects observed on cellular apoptosis and patient’s survival upon selective MEK inhibitor treatment. Within this scenario, it is plausible to hypothesize that combined therapies using both MEK and PI3K inhibitors might display synergistic effects on FOXO3a reactivation, which in turn leads to the restoration of the Sprouty1 levels. Therefore, a fascinating approach based on the AKT inhibitor Uprosertib (GSK2141795) has been applied for solid tumors [47,48]. In AML a phase II trial is ongoing exploring the efficacy of this approach which forecasts the selective blockage of MEK and AKT (ClinicalTrials.gov Identifier: NCT01907815). Different MEK inhibitors have been developed including Trametinib (GSK1120212) and Binimetinib (MEK162). Both have demonstrated activity in different types of solid tumors [49,50]. Trametinib has demonstrated activity also in refractory and relapsed acute myeloid leukemias [51]. Our data identify the FOXO/Sprouty pathway as an effective target in acute myeloid leukemia. Furthermore, the persistence of residual disease during remission is nowadays recognized as a relevant prognostic factor that requires tight monitoring. Consistently, it cannot be excluded that the persistence of residual AML blasts during remission could still harbor the aberrant FoxO3a/Sprouty1 signature. If detectable, such a signature could be a means for measuring small quantities of residual disease, which, in turn, would be useful for assessing who requires additional therapies.

## 5. Conclusions

With the present study, for the first time, we assessed the ole of Sprouty1 in a rather large cohort of adult AML patients, as previously observed for SPRED1 in pediatric leukemia. Moreover, our data indicate that AML blasts exploit RAS activated downstream signaling pathways (MEK-ERK1/2 and PI3K-AKT) to sustain primarily cell survival and proliferation. Furthermore, we speculate that the deregulated Foxo3a/Sprouty1 axis might represent a peculiar signature of AML blasts. Overall this suggests that future therapeutic interventions must take into account to specifically and simultaneously target these pathways.
